# Improved Position Accuracy of Foot-Mounted Inertial Sensor by Discrete Corrections from Vision-Based Fiducial Marker Tracking

**DOI:** 10.3390/s20185031

**Published:** 2020-09-04

**Authors:** Humayun Khan, Adrian Clark, Graeme Woodward, Robert W. Lindeman

**Affiliations:** 1 Human Interface Technology Laboratory, University of Canterbury, Christchurch 8041, New Zealand; gogo@hitlabnz.org; 2 Wireless Research Centre, University of Canterbury, Christchurch 8041, New Zealand; graeme.woodward@canterbury.ac.nz; 3 School of Product Design, University of Canterbury, Christchurch 8041, New Zealand; adrian.clark@canterbury.ac.nz

**Keywords:** foot-mounted inertial sensor, zero-velocity update, fiducial marker tracking, extended Kalman filter, visual-inertial sensor fusion

## Abstract

In this paper, we present a novel pedestrian indoor positioning system that uses sensor fusion between a foot-mounted inertial measurement unit (IMU) and a vision-based fiducial marker tracking system. The goal is to provide an after-action review for first responders during training exercises. The main contribution of this work comes from the observation that different walking types (e.g., forward walking, sideways walking, backward walking) lead to different levels of position and heading error. Our approach takes this into account when accumulating the error, thereby leading to more-accurate estimations. Through experimentation, we show the variation in error accumulation and the improvement in accuracy alter when and how often to activate the camera tracking system, leading to better balance between accuracy and power consumption overall. The IMU and vision-based systems are loosely coupled using an extended Kalman filter (EKF) to ensure accurate and unobstructed positioning computation. The motion model of the EKF is derived from the foot-mounted IMU data and the measurement model from the vision system. Existing indoor positioning systems for training exercises require extensive active infrastructure installation, which is not viable for exercises taking place in a remote area. With the use of passive infrastructure (i.e., fiducial markers), the positioning system can accurately track user position over a longer duration of time and can be easily integrated into the environment. We evaluated our system on an indoor trajectory of 250 m. Results show that even with discrete corrections, near a meter level of accuracy can be achieved. Our proposed system attains the positioning accuracy of 0.55 m for a forward walk, 1.05 m for a backward walk, and 1.68 m for a sideways walk with a 90% confidence level.

## 1. Introduction

Accurately and reliably determining the position and heading of first responders undertaking training exercises can give valuable insight into their situational awareness of the current scenario and provide the context of the decisions made. Measuring movement requires an accurate positioning system. In outdoor environments, the global navigation satellite system (GNSS) is a viable option depending on the availability of satellite signals, but in indoor spaces, these signals can be blocked or have reduced accuracy due to multipath interference. Various indoor positioning systems have been proposed in the past, often using technologies such as wireless infrastructure, inertial and magnetic sensing, ultrasound, and computer vision. These methods either utilise an internal mechanism to measure the change in position and orientation (known as relative positioning systems) or require an external frame of reference to localise (absolute positioning systems) [1].

Existing indoor positioning solutions are either not accurate (e.g., drift in inertial-sensor-based solutions) or require significant energy resources (e.g., SLAM approaches). Inertial-sensor-based solutions assume the initial position and heading are known, and then measure subsequent changes in position and heading values. However, inertial sensors suffer from biases and drift errors which reduce the accuracy substantially as subsequent values are derived from the previous ones. One method for reducing the accumulated effects of errors is by mounting the IMU on the foot of the user [2]. A foot-mounted IMU uses a stand-still state of the foot during different motion types to correct the accrued error since the last stand-still state. This correction mechanism significantly decreases the rate of error accumulation. However, the error becomes significant after a certain distance or with variations in user movement, such as a change in walking speed, walking sideways, or walking backward. To ensure higher overall accuracy of the localisation system, an external correction mechanism by an absolute positioning system (APS) is required. The choice of APS depends on the type of application and its requirements. An application such as virtual reality (VR) using head-mounted displays (HMDs) requires a sub-millimeter level of accuracy and, in general, are not limited by power, computation, or installed infrastructure. Therefore, these systems can use higher-grade APS technology, which only works over a smaller area, such as a desk or a room, and require an active infrastructure.

The current indoor positioning system for training exercises [3,4] require extensive active infrastructure installation which is not viable for exercises taking place in remote areas. Unavailability of power at the training site, short set up time, and the sheer scale of the environment make these systems impractical to deploy. There are only limited APS, namely, fiducial markers [5] and passive RFID tags [6], which uses passive infrastructure and can be calibrated automatically. The passive RFID tags have a limited range, so a vision-based fiducial marker positioning system is more appropriate. Consultation with industry indicated that the training exercises take place over multiple days, and the positioning system should operate without recharging. The participants wear the positioning system; if it uses a lot of energy, then more batteries are required which means more weight to be carried by first responders, and the size of the battery becomes a limitation. If only the vision-based fiducial marker tracking system is used for positioning, it will require a lot of batteries to last multiple days, as typically low-power consuming camera uses 1.6 to 1.8 W [7], which is 16 to 18 times more than the IMU that uses 100 mW [8]. Considering these limitations, we devised a sensor fusion between foot-mounted IMU and vision-based fiducial marker tracking system. For ensuring a long battery life with less computation and sensor resource usage, an adaptive mechanism is described in this paper to use walking motion type (forward walk, sideways walk, and backward walk) as an input to discretely correct for the position and heading errors. With our proposed solution, we can achieve a positioning accuracy of a 0.55 m for forward walk, 1.05 m for backward walk, and 1.68 m for sideways walk with 90% confidence level for a 250 m walking trajectory. Our system proves that we can maintain a high level of localisation accuracy even with discrete fixes from the more accurate APS. In our paper, we aim to answer the following research questions:

- What is the effect of different walking motion types (forward walk, sideways walk, and backward walk) on position and heading error accumulation?
- What is the appropriate distance to trigger corrections from the vision-based fiducial marker tracking system to achieve near sub-meter accuracy?

The paper is structured as follows. In Section 2, we discuss the related research on personal localisation using inertial sensors and vision-based tracking. Towards the end of the section, we also discuss our rationale for choosing a loosely-coupled sensor fusion approach over a tightly-coupled approach. In Section 3, we describe the system design, individual components, and the sensor-fusion approach. In Section 4, we evaluate the performance of our system and provide a comparative analysis with other related research. Lastly, in Section 5, we conclude the paper and outline possible future directions.

## 2. Related Work

### 2.1. Tracking with Inertial Sensors

The simplest approach to get the position and heading from an IMU is by double integration of the acceleration value to get distance, and a single integration of angular velocity to get orientation. However, a small bias or noise in the acceleration value can lead to a much larger position error due to the double integration. This approach only works with “tactical grade” IMUs, which are bulky and expensive [9].

Consumer-grade MEMS-based IMUs use methods based on a human-kinematic model to periodically correct for the position error, zero velocity update (ZUPT) [2,10,11], or empirically establish a formula that correlates the acceleration and angular velocity to change in distance (step length) [12,13]. These methods depend on the mounting location of the IMU on the human body, as each part of the body goes through different dynamics during walking. Most common locations with stable position and heading estimation are the foot and the waist. ZUPT can only be applied if the IMU is mounted on the foot of the user, as it senses the stance phase of the human gait, which can only be accurately determined when foot velocity is near zero [14].

A waist-mounted IMU, on the other hand, empirically relates the step detection and step length estimation with a change in inertial sensor values [13]. This method assumes that the user is always walking forward and not walking sideways or backward, thereby limiting them to be used in only specific applications. Pocket-mounted and handheld devices also use similar approaches as waist-mounted IMUs. In our application, a ZUPT-based method is more relevant as it covers a wider range of motion (walking forward, sideways, and backward, running, crawling and climbing) and results in more-accurate position values [15].

ZUPT methods, however, only result in an accurate localisation when the stance phase (stationary IMU) is detected accurately. The duration of the stance phase is used as a measurement to remove the accumulated error. Stance phase detection is done with an accelerometer, gyroscope, or both sensor data, and there is a range of implementations. Some are based on simple threshold values, while others are statistically derived. Simpler approaches use acceleration magnitude [16], acceleration moving variance [17], or angular-velocity values [18]. Statistical approaches use statistical methods, such as a generalised likelihood ratio test [10], and are more robust and adaptive to changes in motion type. Therefore, we devised our system using a stance hypothesis and an optimal estimation (SHOE) method [10].

### 2.2. Vision-Based Localisation

Vision-based localisation systems have often been used in the past to correct bias and error accumulated by inertial sensors [15,19,20,21]. These systems identify feature points in the surrounding environment and estimate camera pose from the detected feature points which are tracked over multiple frames for position and orientation estimation. Some environments, such as training environments, lack features, but can be mapped prior to localisation. These processes can be made more efficient and robust by placing fiducial markers in the environment. Each fiducial marker can be identified by its unique pattern and placed around the environment to appropriately provide an absolute position and heading value for mobile agents (e.g., the first responders).

Many of the positioning algorithms with sensor fusion between vision-based tracking and inertial sensors use vision as the primary source of localisation, assisted by inertial sensors when vision problems arise (e.g., due to occlusion or fast motion) [22,23,24,25,26]. These systems report the accuracy of a few millimeters. However, they require significant energy resources which do not work for mobile agents with limited battery supply. Therefore, having energy-efficient, foot-mounted inertial sensors as the primary positioning system coupled with discretely used vision-based tracking is a more suitable approach for mobile agents.

### 2.3. Sensor Fusion Approaches

Most of the visual-inertial systems which have vision as the primary source of tracking, the camera and IMU are tightly coupled [27,28]. The tight coupling allows the direct fusion of the raw sensor values, as the sensors are rigidly attached, and there is a fixed transformation between the two sensors. The position and heading values are calculated from the combined raw sensor values. However, in a loosely-coupled system, position and heading values are calculated at each sensor before fusing the data from two sensors [29]. In our case, the camera has to be mounted on the head to give an unobstructed view to the ceiling, and the IMU has to be attached to the foot to calculate accurate position information using ZUPT. Therefore, a loosely-coupled approach results in a more accurate position and heading estimate. In a loosely-coupled system, the position and heading value can be combined by averaging the results (a deterministic approach), or in a probabilistic way (unscented Kalman filter, extended Kalman filter, or particle filter). With a probabilistic approach, each calculated position and heading value from the sensor is given an uncertainty value to describe the accuracy of each system. For our positioning system, we used an extended Kalman filter to combine the position and heading values calculated at each sensor.

## 3. System Design

**Figure 1:**
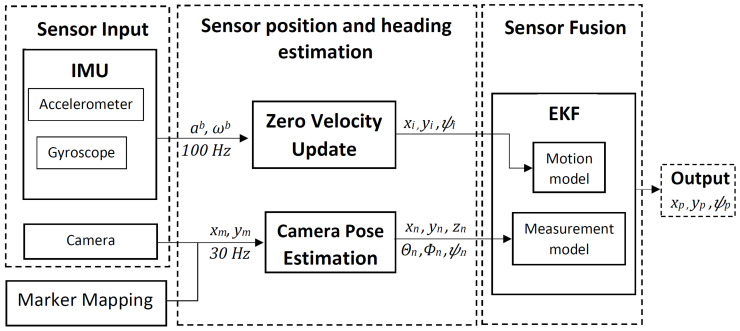
System overview.

This section describes the implementation of our system, which has three components: foot-mounted IMU, fiducial markers, and head-mounted camera. The overview of our system is shown in Figure 1. First, position $\left(x_{i},y_{i}\right)$ and heading $\left(\psi_{i}\right)$ values are calculated from the acceleration $\left(a^{b}\right)$ and angular velocity $\left(\omega^{b}\right)$ of the foot-mounted IMU using ZUPT method, and the whole process is described in Section 3.1. Then, the camera 6 DOF pose $\left(x_{n},y_{n},z_{n},\theta_{n},\phi_{n},\psi_{n}\right)$ is estimated from the fiducial markers value $\left(x_{m},y_{m}\right)$ in world frame of reference. This is covered in Section 3.2. The orientation representation used in our system is Euler angles, roll (θ), pitch (ϕ), and yaw (ψ). The sensor fusion uses pose estimate from both sensors and outputs the position $\left(x_{p},y_{p}\right)$ and heading $\left(\psi_{p}\right)$ of the user. The EKF-based sensor fusion algorithm is described in Section 3.3.

### 3.1. Foot-Mounted IMU

**Figure 2:**
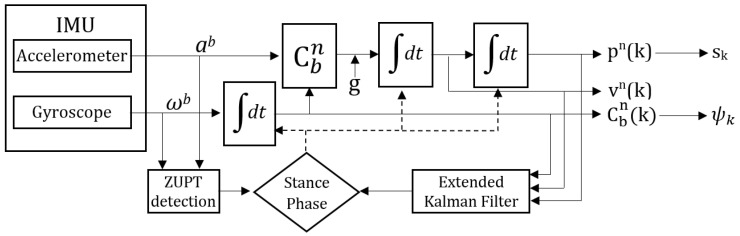
Block diagram of the ZUPT EKF-based correction.

During motion, the human feet alternate between swing (moving) and stance (stationary) phases, and the ZUPT method uses the stance phase to correct the accumulated error. The stance phase detection of our system is based on Skog et al.’s work [10], which uses three inputs: acceleration magnitude, acceleration variance, and angular velocity. ZUPT is implemented with an extended Kalman filter (EKF), and the error in position ($p^{n}$), velocity ($v^{n}$), and orientation ($C_{b}^{n}$) values are fed back into the EKF to rectify their values. The *n* denotes the navigation frame of reference, and *b* signifies the body frame of reference. The stance phase is also used to calculate step length, $s_{k}$, and step heading, $\psi_{k}$. The next state values are calculated based on the frequency of the IMU with Equations (1)–(4), where *k* is the sample index, $dt_{k}$ is the time difference since the last update, $a^{b}$ is the acceleration value from the accelerometer measured in the body frame of reference, $\omega^{b}\left(\omega_{x}^{b},\omega_{y}^{b},\omega_{z}^{b}\right))$ is the angular velocity from the gyroscope measured in the body frame of reference and *g* is the gravity. The foot orientation values at sample k are roll, $\theta_{k}=arctan\left(C_{b}^{n}\left(3,2\right),C_{b}^{n}\left(3,3\right)\right)$, pitch, $\phi_{k}=-arcsin\left(C_{b}^{n}\left(3,1\right)\right)$, and yaw, $\psi_{k}=arctan(C_{b}^{n}\left(2,1\right),C_{b}^{n}\left(1,1\right)$. Figure 2 illustrates the whole process:

(1) $$p^{n}\left(k+1\right)=p^{n}\left(k\right)+\frac{\left(v^{n}\left(k\right)+v^{n}\left(k+1\right)\right)}{2}*dt_{k}$$

(2) $$v^{n}\left(k+1\right)=v^{n}\left(k\right)+\left(C_{b}^{n}\left(k\right)*a^{b}\left(k+1\right)-g\right)*dt_{k})$$

(3) $$C_{b}^{n}\left(k+1\right)=C_{b}^{n}\left(k\right)*\left[\left(\omega^{b}\left(k+1\right)\times \right)*dt_{k}\right]$$

(4) $$\left[\omega^{b}\left(k\right)\times \right]=\left[\begin{array}{ccc} 0 & -\omega_{z}^{b}\left(k\right) & \omega_{y}^{b}\left(k\right)\\ \omega_{z}^{b}\left(k\right) & 0 & -\omega_{x}^{b}\left(k\right)\\ -\omega_{y}^{b}\left(k\right) & \omega_{x}^{b}\left(k\right) & 0 \end{array}\right]$$

**Figure 3:**
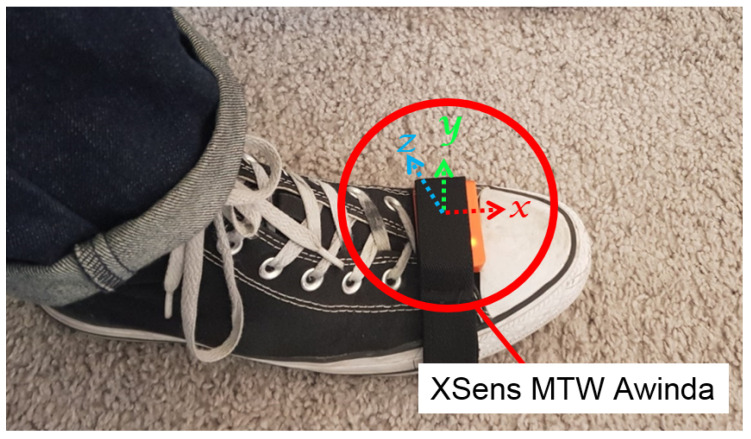
XSens MTW Awinda attached to the foot of the user, and the IMU’s body frame of reference.

Initially, low-cost TDK, Bosch, and STMicroelectronics IMUs were considered for our system; however, the past research by Wagastaff et al. [30] and IMU specifications [8,31,32,33] indicated higher noise in accelerometer and gyroscope signals; therefore, we decided to use XSens MTwAwinda IMU. The IMU was strapped to the user’s foot, as shown in Figure 3, and it was wirelessly connected to a laptop carried by the user, which recorded the inertial sensor data along with the timestamp.

### 3.2. Vision-Based Fiducial Marker Tracking

**Figure 4:**
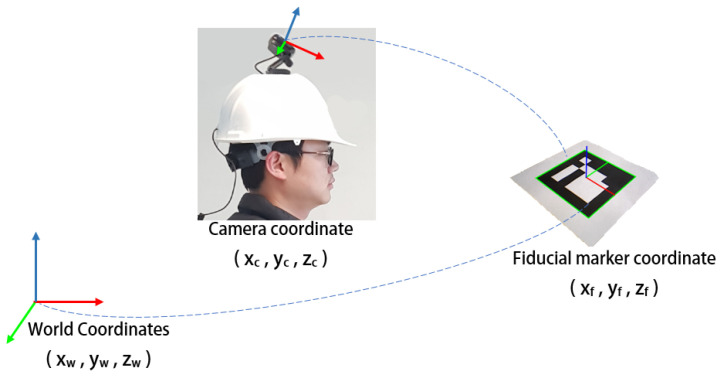
Computing camera pose from fiducial markers and translating to the world coordinates.

The process to calculate the user position is illustrated in Figure 4. In our positioning system, the fiducial markers were installed on the ceiling of the HITLab NZ building. Each marker had a unique identification and a measured coordinate value with respect to the world origin. The coordinate values were measured using a Bosch PLR 50C laser measure, a Bosch three-point laser, and a Leica surveyor tripod. We used OpenCV ArUco fiducial markers for our system [34]. Camera pose was estimated using the transformation matrix between the marker coordinate system and camera coordinate system. As the marker’s coordinate system had a known transformation with respect to the world coordinate system, the camera’s position and orientation could be determined relative to the world coordinate system.

### 3.3. Loosely-Coupled Sensor Fusion

In the proposed algorithm, the foot-mounted IMU and head-mounted camera are loosely coupled using EKF-based sensor fusion. The motion model of the EKF is driven by the foot-mounted inertial sensor data, and the measurement model by the vision-based fiducial marker detection. The state vector, $x_{k}$, of the system is a 2D position $\left(px_{k},py_{k}\right)$ and heading $\left(\psi_{k}\right)$ of the user, and is described by the equation, $x_{k}={\left(px_{k},py_{k},\psi_{k}\right)}^{T}$ where *T* is the transpose of the matrix and *k* is the sample at time, $t_{k}$. The motion model is propagated with the function, $x_{k+1}=g\left(x_{k},u_{k},w_{k}\right)$, which is given by the equation:

(5) $$x_{k}=x_{k-1}+{\left(s_{k}*cos\left(\psi_{k}\right),s_{k}*sin\left(\psi_{k}\right),\psi_{k}\right)}^{T}+w_{k}$$

where $w_{k}$ is the motion model noise. The $s_{k}$ is the step length and is computed using the ZUPT approach described in Section 3.1. The covariance matrix, $P_{k}$, is propagated using the Jacobian matrix, $G_{k}$, and the motion model noise matrix, $Q=E(w_{k}*w_{k}^{T})$. The $G_{k}$ is given by the Equation (7), and *Q* is computed with the accelerometer and gyroscope noise values. The equation for covariance matrix propagation is as follows:

(6) $$P_{k}=G_{k}*P_{k-1}*G_{k}^{T}+Q_{k}$$

(7) $$G_{k}=\left(\begin{array}{ccc} 1 & 0 & -s_{k}*sin\left(\psi_{k}\right)\\ 0 & 0 & s_{k}*cos\left(\psi_{k}\right)\\ 0 & 0 & 1 \end{array}\right)$$

The measurement model is updated from the fiducial marker system and is propagated with the function, $z_{k}=h\left(x_{k},v_{k}\right)$, where $v_{k}$ is measurement noise, which is used to calculate the measurement noise covariance matrix, $R_{k}=E\left(v_{k}*v_{k}^{T}\right)$. The state and covariance matrices in the measurement model are propagated with the following equations:

(8) $$x_{k+1}=x_{k}+K_{k}*\left(z_{k+1}-h\left(x_{k}\right)\right)$$

(9) $$P_{k+1}=\left(I-K_{k}*H_{k}\right)P_{k}$$

where $K_{k}$ is the Kalman gain and can be calculated by Equation (11). The $H_{k}$ is the Jacobian matrix of function $h(x_{k})$, and it is calculated by the partial derivative of $h(x_{k})$ with respect to state *x*. It is evaluated using the prior state estimate $x_{k-1}$, and is given by the equation as follows:

(10) $$H_{k}=\frac{\partial h}{\partial x}|x=x_{k-1}$$

(11) $$K_{k}=P_{k}*H_{k}^{T}{\left(H_{k}*P_{k}*H_{k}+R_{k}\right)}^{-1}$$

**Figure 5:**
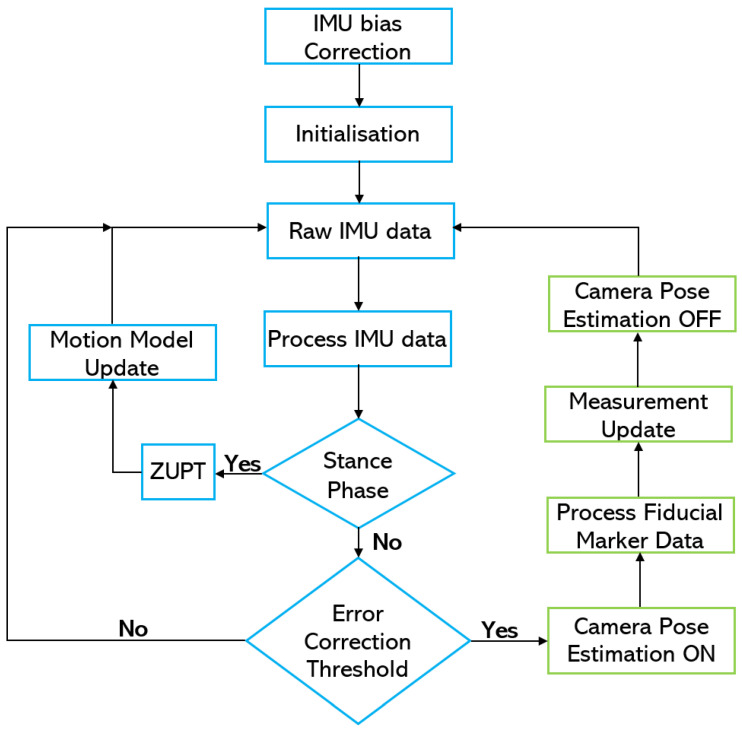
Sensor-fusion flow chart.

The IMU is calibrated by removing the accelerometer and gyroscope bias calculated from the acceleration and angular velocity values recorded during the initial 10 s. The user remains still during this time. The position and heading values of the system are also initialised from the fiducial marker system. After these two steps, the foot-mounted IMU computes the change in step length and adds it to the last position value. Similarly, the heading value is also tracked by keeping track of the last change in orientation value. However, when the error threshold is reached, the camera pose estimation part of the algorithm is initiated, and the measurement model is applied. The estimated camera pose is translated into position and heading values, which in turn are used to correct the system’s position and heading values. The camera-pose estimation method is deactivated after the update. This process is repeated whenever the error threshold is reached. The flowchart in Figure 5 illustrates the implementation of our system.

## 4. Evaluation

### 4.1. Experiment Setup

**Figure 6:**
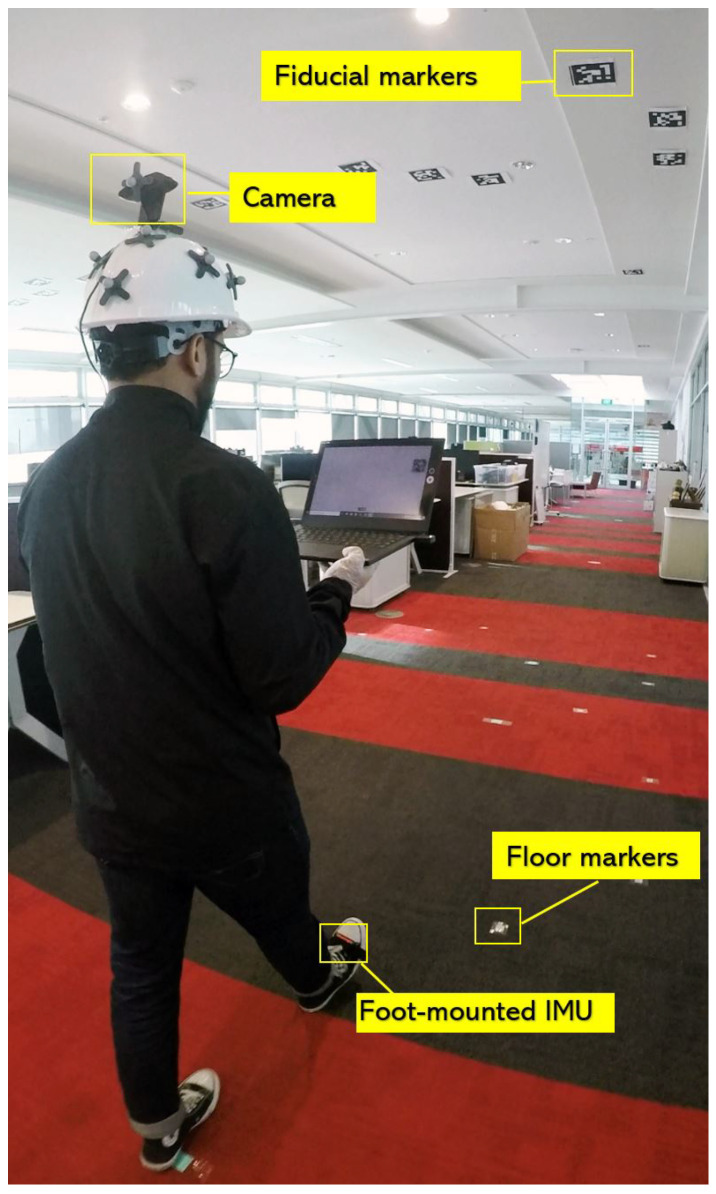
System setup.

The sensors worn by the user consisted of an XSens MTW Awinda IMU on the right foot and a head-mounted Logitech C920 web camera facing the ceiling of the building. Figure 6 shows the sensor setup. Both sensors recorded data on the laptop carried by the user. The IMU recorded data at 100 Hz, and the camera captured video at 30 fps with 1080 p resolution. An initial 10 s of data was recorded with the user in a stationary state to remove the accelerometer and gyroscope bias. The bias removal was done before each walk. The data from the IMU had three-dimensional acceleration, three-dimensional angular velocity, and timestamp values. The data from the head-mounted camera video contained the fiducial marker ID along with the camera pose and timestamp values.

**Figure 7:**
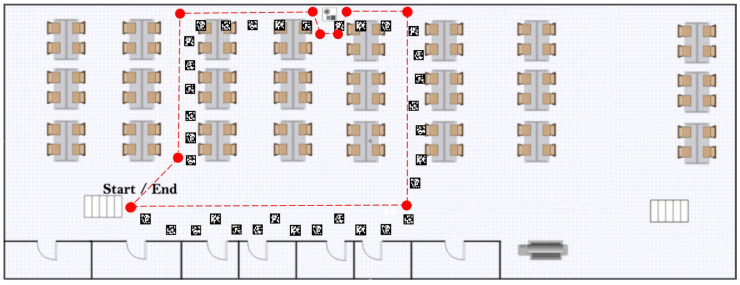
Planned indoor walking trajectory.

To model the error accumulated by each motion type and measure the accuracy of the positioning system, an approximately 50 m long walking trajectory was planned under the installed ceiling markers inside the HIT Lab NZ building. The planned trajectory is shown in Figure 7 along with the installed markers.

### 4.2. Ground Truth

For ground truth, a similar approach to Fusco et al. [20,35] was adopted. The tracked user was followed by a person who recorded a video from an external camera. The video recorded the position of the user’s feet which could later be measured with reference to markers placed on the floor at 0.5 m intervals, as well as to the screen of the laptop the user was holding, showing the ceiling fiducial marker video. The observed feet position was measured using a Bosch PLR 50C laser measure. Ground truth videos were recorded with a GoPro Hero5 camera and were analysed offline to compute the Euclidean distance error between the ground truth and the positioning system estimated position. The ground truth error was conservatively estimated to be 0.5 m based on the installed floor markers. With a similar approach, Wagstaff et al. were able to achieve 0.31 m ground truth accuracy [30].

### 4.3. Experiment

The experiment consisted of three conditions, forward walk, sideways walk and backward walk. For each motion type, five loops were done of the planned trajectory, resulting in a 250 m long trajectory for each walk type. Each motion type was repeated three times to have a better understanding of the error accumulation. Fiducial marker tracking was also performed, which was to be later fused with the ZUPT-based IMU localisation system. The three repetitions were meant to observe a pattern of position and heading error for each motion type. The experiment was carried out by one person walking at a normal pace (about 1 step per second) along the planned trajectory.

### 4.4. Results and Discussion

#### 4.4.1. Foot-Mounted IMU Trajectories

**Figure 8:**
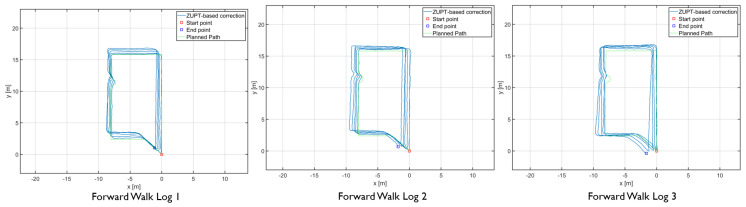
Forward walk trajectories of the foot-mounted IMU.

**Figure 9:**
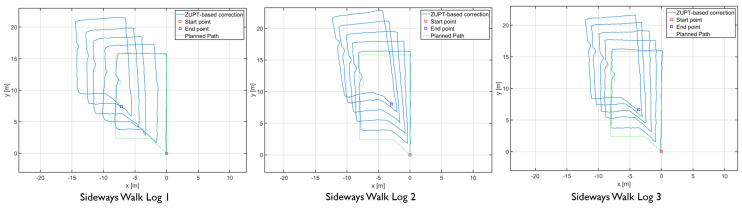
Sideways walk trajectories of the foot-mounted IMU.

**Figure 10:**
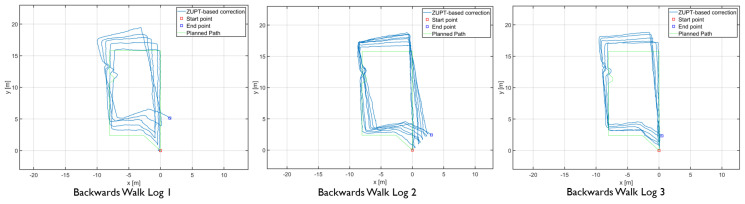
backward walk trajectories of the foot-mounted IMU.

The results showed differences in the amount of position and heading error accumulation for each motion type. The three trajectory plots of each repetition of forward, sideways, and backward walk can be seen in Figure 8, Figure 9 and Figure 10. These plots also help us to answer our first research question. From the trajectory plots, we can observe that the position and heading values for the forward walk were closer to the ground-truth trajectory. However, the position value after each 50 m loop drifted which indicated a scaling error. The position value at the end of the trajectories drifted by 1.59 m on average. Sideways walk trajectory plots for all three repetitions showed a scaling error which resulted in a noticeable position drift after each loop. The heading values in the sideways walk trajectories (Figure 9) were closer to the walked trajectory. The mean drift in final position value for the sideways walk was 8.65 m. For the backward walk trajectories, a large drift in heading value was noticed after every 25 m, which lead to a sizeable error in the position values. The mean drift in final position value for the backward walk was 3.69 m. From the sideways walk and backward walk trajectories, we observed a noticeable increase in either position or heading values after 25 m. Therefore, we devised a correction mechanism from the fiducial marker position system at 25 m, 50 m, 75 m and 100 m intervals, and compared the average error in each case.

#### 4.4.2. Vision-Based Fiducial Marker Trajectories

**Figure 11:**
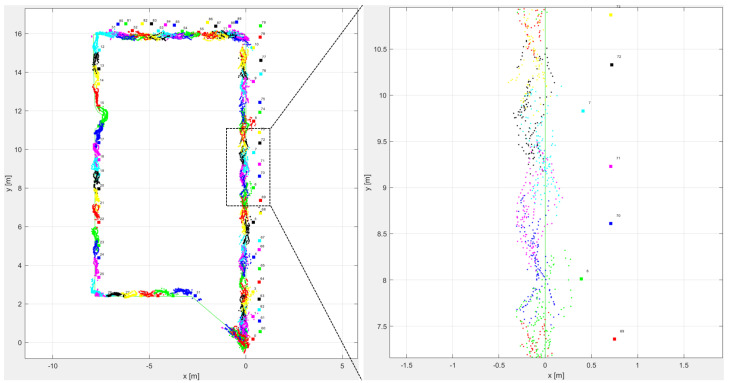
Fiducial marker forward walk trajectory with the zoomed section on the right.

The recorded trajectory plot of the forward walk for fiducial marker tracking is shown in Figure 11. Each marker on the plot is assigned a separate colour, and the detected position by the head-mounted camera has the same corresponding colour. A zoomed-in version of the detected points can be seen in Figure 11 as well. The detected range of the marker was about 3 m, and the observed average accuracy of the detected points was 0.5 m.

#### 4.4.3. Sensor Fusion Results

**Figure 12:**
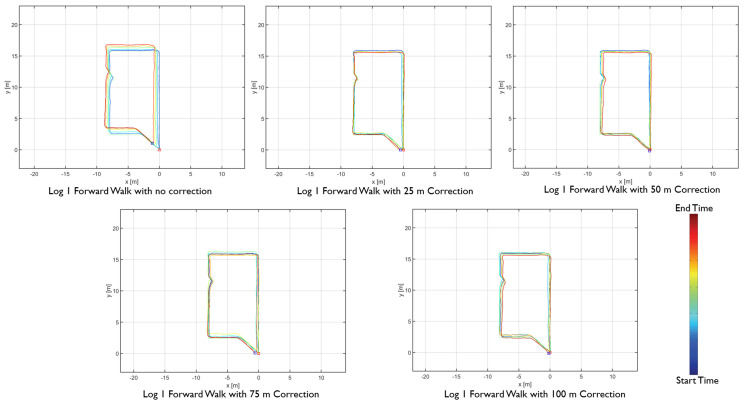
Forward walk distance-based sensor fusion trajectories.

**Figure 13:**
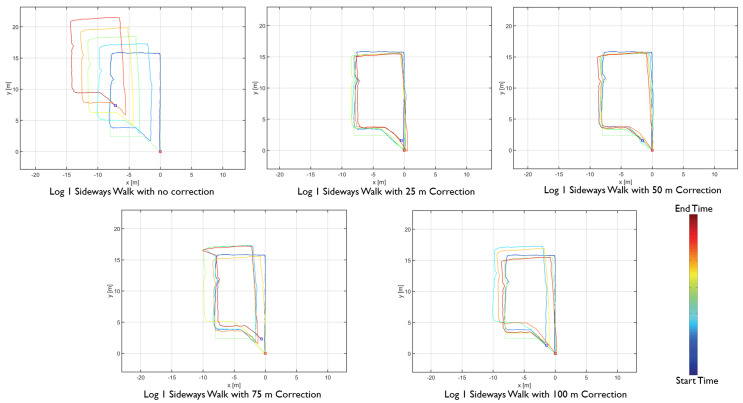
Sideways walk distance-based sensor fusion trajectories.

**Figure 14:**
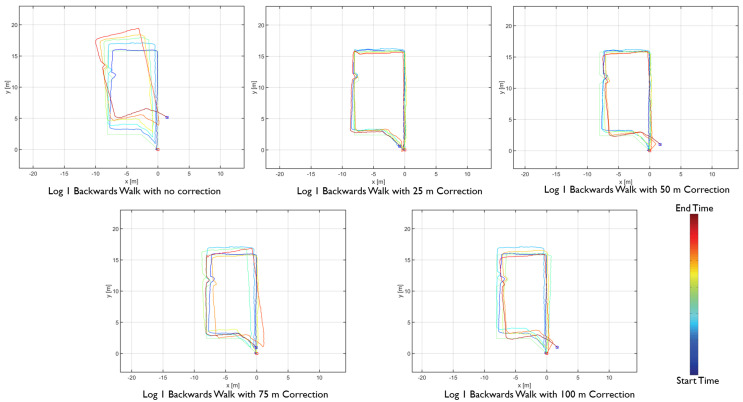
backward walk distance-based sensor fusion trajectories.

**Figure 15:**
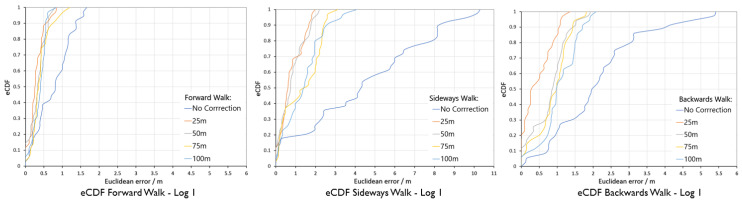
Error eCDF for forward, sideways and backward walk.

The proposed distance-based correction was done for all three walking motion types, forward walk, sideways walk, and backward walk at 25 m, 50 m, 75 m and 100 m intervals. The total distance walked for each motion type was 250 m. Trajectories resulting from the sensor fusion are shown in Figure 12, Figure 13 and Figure 14. From the empirical CDF plots, shown in Figure 15, and Table 1, we can observe that a near sub-meter accuracy can be achieved with our proposed sensor fusion solution for a forward and backward walk. However, for the sideways walk, a shorter correction distance is required to improve the position accuracy.

With the results obtained, we can answer our second research question. The appropriate distance to achieve near sub-meter accuracy for forward walking motion type is 100 m as 90% of the Euclidean distance error was 0.55 m. For backward walk, the correction distance is 25 m with 90% of the Euclidean distance error was 1.05 m. For the sideways walk, our system achieved below 2 m Euclidean error with 25 m corrections. For near sub-meter accuracy, a shorter distance correction method is needed.

#### 4.4.4. Comparative Analysis with Recent Work

This section covers a comparative analysis of our work with some of the recent research works which have used IMU and infrastructure based sensor fusion approach. Due to the variation in total distance travelled by each research, we calculated the error percentage of the travelled distance. Table 2 shows the comparison of our system with similar recent work; it includes the system description of the positioning system, the motion types detected by the system, the total distance travelled during the experiment and the error percentage.

When we compare our results in Table 2 with similar approaches, we find that our system is comparatively accurate. However, when we calculate the percentage error of the forwards, sideways and backward walk with no correction, which is the case for [15,30,36,37]. It provides a comparable metric with forward walk 0.55% error, sideways walk 3.28% error, and backward walk 1.62% error. With the passive infrastructure correction from the fiducial markers, the overall system accuracy became better than [15,30,36,39], or comparable to [37,38], but increasing the overall power consumption of the system which in turn implies shorter battery life. The overall power consumption of our system is 0.89 W. The hybrid foot-mounted IMU and UWB system performed to a similar degree when we look at error to distance covered percentage; however, the data provided are only for a short walk 26 m long. A longer walk test will provide more substantial evidence. In addition, the UWB system requires an active power supply at the infrastructure, which makes it unviable for training exercise application. The sensor fusion between foot-mounted IMU and WiFi fingerprinting-based positioning system has relatively lower accuracy due to the variation of access points available in the environment, which is also indicated by the author. The choice of positioning technology with passive infrastructure is limited; however, the positioning results of our system indicate that it can potentially be considered for applications which lack the active power infrastructure or are battery limited.

## 5. Conclusions and Future Work

We present a novel sensor fusion approach which uses motion types and estimated distance travelled to discretely correct a foot-mounted inertial positioning system with a more accurate vision-based fiducial marker tracking system. The motion types considered for our system are variation in walking movement, which are walking forward, walking sideways and walking backward. We characterised the error accumulated for each motion type and used it to configure our positioning system. Our sensor fusion approach preserves the position and heading accuracy without having continuous input from the absolute positioning system. In the future, we will quantify power consumption by each component of the positioning system and optimise the overall energy efficiency of the system. We will also explore other ways of determining when to initiate use of the vision-based system, such as estimated error or other dynamic events that arise during the walk. Finally, it should be noted that our goal is to evaluate systems for after-action review, as opposed to tracking people in real-time. However, many of the findings we make in our work can be applied to real-time tracking. We will explore these more in the future.

## Figures and Tables

**Table 1:** Localisation error for the 90% of eCDF value for the 250 m walk of each motion type.

Walk Type	No Correction	25 m	50 m	75 m	100 m
Forward Walk	1.37 m	0.55 m	0.61 m	0.75 m	0.55 m
Sideways Walk	8.20 m	1.68 m	1.79 m	2.42 m	2.55 m
backward Walk	4.04 m	1.05 m	1.43 m	1.43 m	1.70 m

**Table 2:** Localisation error and method comparison with recent work.

Authors	Ref.	System Description	Motion Types	Total Dist. (m)	Error %
Our System	NA	Foot-mounted IMU and vision-based fiducial marker tracking	Forward, Sideways, and backward Walking	250 m	0.44%
Shi et al.	[36]	Foot-mounted IMU	Forward Walking and Running	100 m, 400 m	1.06%
Qiu et al.	[37]	Foot-mounted IMU	Forward Walking and Climbing	50 m, 425 m	0.35%
Wagstaff et al.	[30]	Foot-mounted IMU	Forward Walking and Running	130 m	2.47%
Rantanen et al.	[15]	Foot-mounted IMU	Forward Walking, Running, and Climbing	170 m	2.64%
Chen et al.	[38]	Foot-mounted IMU and UWB-based positioning system	Walking	26 m	0.62%
Chen et al.	[39]	Handheld IMU device and WiFi fingerprinting-based positioning system	Walking	90 m, 100 m, and 260 m	1.89%

## Abbreviations

The following abbreviations are used in this manuscript:

IMU	Inertial Measurement Unit
EKF	Extended Kalman Filter
ZUPT	Zero Velocity Update
APS	Absolute Positioning System
GNSS	Global Navigation Satellite System
MEMS	Micro-Electro-Mechanical Systems
SLAM	Simultaneous Localization and Mapping
SHOE	Stance Hypothesis and Optimal Estimation
CDF	Cumulative Distribution Function
eCDF	Empirical Cumulative Distribution Function

## References

[1] Woodman O. (2010). Pedestrian Localisation for Indoor Environments. Ph.D. Thesis.

[2] Foxlin E. (2005). Pedestrian tracking with shoe-mounted inertial sensors. IEEE Comput. Graph. Appl..

[3] PLUS Location Systems The PLUS Activate Platform. https://pluslocation.com/solutions/.

[4] Ubisense Ubisense: Dimension4 UWB RTLS. https://ubisense.com/dimension4/.

[5] Muñoz-Salinas R., Medina-Carnicer R. (2020). UcoSLAM: Simultaneous localization and mapping by fusion of keypoints and squared planar markers. Pattern Recognit..

[6] Seco F., Jimenez A.R. Autocalibration of a wireless positioning network with a FastSLAM algorithm. Proceedings of the International Conference on Indoor Positioning and Indoor Navigation (IPIN).

[7] Chen X., Nixon K.W., Chen Y. Practical power consumption analysis with current smartphones. Proceedings of the 29th IEEE International System-on-Chip Conference (SOCC).

[8] XSens Technologies (2019). MTi 1-Series Datasheet, Document MT0512P.

[9] VectorNav Technologies Inertial Measurement Units and Inertial Navigation. https://www.vectornav.com/support/library/imu-and-ins.

[10] Skog I., Handel P., Nilsson J.O., Rantakokko J. (2010). Zero-velocity detection—An algorithm evaluation. IEEE Trans. Biomed. Eng..

[11] Jimenez A.R., Seco F., Prieto C., Guevara J. A comparison of pedestrian dead-reckoning algorithms using a low-cost MEMS IMU. Proceedings of the IEEE International Symposium on Intelligent Signal Processing.

[12] Alvarez J.C., Alvarez D., López A., González R.C. (2012). Pedestrian navigation based on a waist-worn inertial sensor. Sensors.

[13] Weinberg H. (2002). Using the ADXL202 in pedometer and personal navigation applications. Anal. Devices AN-602 Appl. Note.

[14] Díez L.E., Bahillo A., Otegui J., Otim T. (2018). Step length estimation methods based on inertial sensors: A review. IEEE Sens. J..

[15] Rantanen J., Mäkelä M., Ruotsalainen L., Kirkko-Jaakkola M. Motion context adaptive fusion of inertial and visual pedestrian navigation. Proceedings of the 2018 International Conference on Indoor Positioning and Indoor Navigation (IPIN).

[16] Godha S., Lachapelle G. (2008). Foot mounted inertial system for pedestrian navigation. Meas. Sci. Technol..

[17] Kwakkel S., Lachapelle G., Cannon M. GNSS aided in situ human lower limb kinematics during running. Proceedings of the 21st International Technical Meeting of the Satellite Division of the Institute of Navigation (ION GNSS 2008).

[18] Feliz Alonso R., Zalama Casanova E., Gómez García-Bermejo J. (2009). Pedestrian tracking using inertial sensors. J. Phys. Agents.

[19] Neges M., Koch C., König M., Abramovici M. (2017). Combining visual natural markers and IMU for improved AR based indoor navigation. Adv. Eng. Inform..

[20] Fusco G., Coughlan J.M. Indoor localization using computer vision and visual-inertial odometry. Proceedings of the 2018 International Conference on Computers Helping People with Special Needs(ICCHP).

[21] Mäkelä M., Rantanen J., Kirkko-Jaakkola M., Ruotsalainen L. (2018). Context recognition in infrastructure-free pedestrian navigation—Toward adaptive filtering algorithm. IEEE Sens. J..

[22] Liu H., Zhang G., Bao H. Robust keyframe-based monocular SLAM for augmented reality. Proceedings of the 2016 IEEE International Symposium on Mixed and Augmented Reality (ISMAR).

[23] Babu B.W., Kim S., Yan Z., Ren L. *σ*-dvo: Sensor noise model meets dense visual odometry. Proceedings of the 2016 IEEE International Symposium on Mixed and Augmented Reality (ISMAR).

[24] Schöps T., Engel J., Cremers D. Semi-dense visual odometry for AR on a smartphone. Proceedings of the 2014 IEEE international symposium on mixed and augmented reality (ISMAR).

[25] Yan Z., Ye M., Ren L. (2020). Dense Visual SLAM with Probabilistic Surfel Map. U.S. Patent.

[26] Engel J., Koltun V., Cremers D. (2017). Direct sparse odometry. IEEE Trans. Pattern Anal. Mach. Intell..

[27] Li P., Qin T., Hu B., Zhu F., Shen S. Monocular visual-inertial state estimation for mobile augmented reality. Proceedings of the 2017 IEEE International Symposium on Mixed and Augmented Reality (ISMAR).

[28] Li M., Kim B.H., Mourikis A.I. Real-time motion tracking on a cellphone using inertial sensing and a rolling-shutter camera. Proceedings of the 2013 IEEE International Conference on Robotics and Automation.

[29] Ahmed D.B., Diaz E.M. (2017). Loose coupling of wearable-based INSs with automatic heading. Sensors.

[30] Wagstaff B., Peretroukhin V., Kelly J. Improving foot-mounted inertial navigation through real-time motion classification. Proceedings of the 2017 International Conference on Indoor Positioning and Indoor Navigation (IPIN).

[31] STMicroelectronics (2015). LSM9DS1 Datasheet, Document DocID025715.

[32] TDK InvenSense (2017). ICM-20948 Datasheet, Document DS-000189.

[33] Bosch (2016). BNO055 Datasheet, Document BST-BNO055-DS000-14.

[34] Romero-Ramirez F.J., Muñoz-Salinas R., Medina-Carnicer R. (2018). Speeded up detection of squared fiducial markers. Image Vis. Comput..

[35] Villien C., Frassati A., Flament B. Evaluation of An Indoor Localization Engine. Proceedings of the 2019 International Conference on Indoor Positioning and Indoor Navigation (IPIN).

[36] Shi L.F., Zhao Y.L., Liu G.X., Chen S., Wang Y., Shi Y.F. (2018). A robust pedestrian dead reckoning system using low-cost magnetic and inertial sensors. IEEE Trans. Instrum. Meas..

[37] Qiu S., Wang Z., Zhao H., Qin K., Li Z., Hu H. (2018). Inertial/magnetic sensors based pedestrian dead reckoning by means of multi-sensor fusion. Inf. Fusion.

[38] Chen P., Kuang Y., Chen X. (2017). A UWB/improved PDR integration algorithm applied to dynamic indoor positioning for pedestrians. Sensors.

[39] Chen J., Ou G., Peng A., Zheng L., Shi J. (2018). An INS/WiFi indoor localization system based on the Weighted Least Squares. Sensors.

